# The Significance of Cross-Sectional Shape Accuracy and Non-Linear Elasticity on the Numerical Modelling of Cerebral Veins under Tensile Loading

**DOI:** 10.3390/biology13010016

**Published:** 2023-12-27

**Authors:** Fábio A. O. Fernandes, Clara I. C. Silveira

**Affiliations:** 1 TEMA—Centre for Mechanical Technology and Automation, Department of Mechanical Engineering, University of Aveiro, Campus Universitário de Santiago, 3810-193 Aveiro, Portugal; 2 LASI—Intelligent Systems Associate Laboratory, 4800-058 Guimarães, Portugal; 3 Department of Physics, University of Aveiro, Campus Universitário de Santiago, 3810-193 Aveiro, Portugal

**Keywords:** bridging veins, finite element analysis, geometric accuracy, constitutive model, hyperelastic, Marlow, reduced polynomial

## Abstract

**Simple Summary:**

Traumatic brain injuries, a primary global health concern, can lead to severe disabilities. Acute subdural haematoma is one type of traumatic brain injury resulting from the rupture of bridging veins in the brain. Existing numerical models often oversimplify these structures. This study’s main goals were to understand how the cerebral vein’s cross-sectional shape affects its response, more akin to real-life testing, and to introduce a hyperelastic model for these veins that captures their non-linear behaviour. The results reveal that the vein’s shape significantly influences its response to stretching, highlighting the need for more realistic geometries in modelling. Additionally, hyperelastic models, like the Marlow and polynomial models, were successfully implemented. This research provides valuable insights into the mechanical properties of bridging veins and their response to tensile loading, potentially improving our understanding of traumatic brain injuries.

**Abstract:**

Traumatic brain injury (TBI) is a serious global health issue, leading to serious disabilities. One type of TBI is acute subdural haematoma (ASDH), which occurs when a bridging vein ruptures. Many numerical models of these structures, mainly based on the finite element method, have been developed. However, most rely on linear elasticity (without validation) and others on simplifications at the geometrical level. An example of the latter is the assumption of a regular cylinder with a constant radius, or the geometry of the vein acquired from medical images. Unfortunately, these do not replicate the real conditions of a mechanical tensile test. In this work, the main goal is to evaluate the influence of the vein’s geometry in its mechanical behaviour under tensile loading, simulating the real conditions of experimental tests. The second goal is to implement a hyperelastic model of the bridging veins where it would be possible to observe its non-linear elastic behaviour. The results of the developed finite element models were compared to experimental data available in the literature and other models. It was possible to conclude that the geometry of the vein structure influences the tensile stress–strain curve, which means that flattened specimens should be modelled when validating constitutive models for bridging veins. Additionally, the implementation of hyperelastic material models has been verified, highlighting the potential application of the Marlow and reduced polynomial (of fourth and sixth orders) constitutive models.

## 1. Introduction

Globally, traumatic brain injury (TBI), known as the ‘silent epidemic’, is a leading cause of health problems and disabilities. The annual incidence of TBI is estimated to range from 27 to 69 million worldwide [1,2]. TBI can be defined as a change in brain function or another sign of pathology brought by an external force [3]. Numerous survivors of TBI remain with severe disabilities such as cognitive deficits, sensory processing problems, and behaviour and mental health complications.

One of the most lethal forms of TBI is the acute subdural haematoma (ASDH) which develops when a blood vessel in the subdural space ruptures, forming a blood clot that pressures the brain and ultimately damages it [4,5,6]. The blood vessels found in the subdural space are called bridging veins (BVs), which are delicate and small. Venous anatomy is complex and can be divided into two groups: the superficial medullary or subcortical venous system, and the deep medullary venous system [7]. These groups are defined according to whether blood is diverted to the superficial veins or to the deep cerebral veins and, ultimately, the vein of Galen.

Depending on their location, all cerebral veins typically drain into the nearest dural sinus. The superior dorsal veins drain into the Superior Sagittal Sinus (SSS), the posterior veins and veins of the cerebellum drain into the transverse sinus, and the anterior veins may drain to the SSS, cavernous sinus, or superficial middle cerebral vein. The SSS is the major component of the superficial cerebral venous system. It is a midline venous sinus without valves or muscular tissue in its constitution and courses along the falx cerebri, draining many of the cerebral structures surrounding it [8].

BVs penetrate the arachnoid and dura mater and connect remote parts of the brain with the SSS. The inflow of blood coming from bridging veins into the SSS can be described in five categories: antegrade (along with the flow of the SSS), perpendicular, retrograde (opposed to the flow direction of the SSS), hairpin shaped (changing direction shortly before entering SSS), and lacunae (enlarged venous space) [9].

In terms of size, BVs’ diameter goes from 0.5 to 5.3 mm, changing according to the location along the vein. In the subarachnoid course, the diameter stays more or less the same. Still, as the vein approaches the SSS, the diameter increases and narrows again just before the SSS on the outflow cuff segment, creating a ‘puffy-looking’ vein [10].

Vascular walls comprise 70% water and 30% dry mass like collagen, elastic, proteoglycan, and vascular cells [11] and are divided into three layers intercalated by elastic membranes. The outermost layer, called tunica adventitia, comprises fibroblasts and elastin, but mainly collagen fibres oriented longitudinally as wavy bundles. Tunica media is the middle layer composed of circumferentially aligned smooth muscle cells (SMCs), elastin, and collagen fibres. The innermost layer, tunica intima, is formed of endothelial cells. The thickness of each layer depends on the calibre of the vessel. Medium and small vessels exhibit less stretchability than larger vessels because they have more SMCs and less elastic tissue. Veins contain less elastic tissue and a higher amount of collagen than arteries. At lower pressures (as in the brain), the mechanical behaviour is dominated by the elastic components, which are less stiff [11].

In the BVs, the distribution and orientation of the collagen fibres change significantly according to the location. The subarachnoid portion has a denser structure, and the collagen fibres show a loose webbing pattern in the subdural portion. The collagen fibres are mainly oriented in the longitudinal direction but are circumferential or helicoidal in the outflow cuff segment. This circumferential orientation of the fibres in the outflow cuff segment limits the diameter to a certain size, giving a dynamic resistance against sudden changes in blood flow [10].

Finite element (FE) head models (FEHMs) are a flexible and ethical way of predicting head injuries and their outcomes. Additionally, after thorough validation, FE models can be employed to design several devices, particularly in the optimisation stage, such as protective equipment or medical devices. Various models have been developed over the years, but many lack the presence of cerebral vasculature, including BVs [12,13,14,15]. More recently, some models have included BVs, albeit typically employing solely linear elasticity and using discrete beam or linear spring elements, without adequate validation of the BV model [16,17,18]. BVs are challenging to simulate because of their reduced size, structure, and histology, which is still a hot research topic with constant discoveries.

The geometric accuracy of the BVs’ structure has been investigated through their incorporation in FEHMs, first with solid FE elements (and solid structure) [19] and later with shell elements by taking into account the thickness variability across the BVs’ length [20]. Costa et al. [20] developed and validated a realistic 3D model of BVs and SSS, including the radius between both tubular structures, implementing a damage model that predicted SDH with the highest success in the literature [21,22] by simulating Depreitere’s experiment [23]. Nevertheless, this model was highly dependent on the “anchoring” regions of the BVs [23]. In addition, Costa et al. [20] validated single BVs under tensile loading up to failure, as reported in the experiments of Monea et al. [24]. This validation consisted of the comparison of the stress–strain behaviour until failure. Nevertheless, Costa et al. [20] modelled the geometry as an inflated vessel instead of collapsed, which means the cross-sectional area was correct but not its shape. Additionally, BVs were modelled as elastoplastic, although their non-linear elastic behaviour has been reported [10,25,26,27,28,29]. Nevertheless, in the data reported by Monea et al. [24], by analysing the stress–strain curves, it is evident that damage is present for a significant strain portion. This has also been recently reported by other researchers [30]. Nevertheless, the most significant component is the non-linear elastic one [26,27,30]. Recently, with a similar structural model of BVs implemented in a finite element head model (FEHM) like in [20], the authors modelled the cerebral vasculature (third-order Ogden model), but the BV model was not validated according to mechanical testing data prior to implementation in the FEHM [31].

The Costa et al. [20] model is viable for further use in studies where the main goal is to predict SDH and similar outcomes by simulating the rupture of the veins. However, this model is not ideal for studies where the principal focus is to understand the non-linear elasticity or there is a high dependency on this behaviour. Another factor to consider when looking into the study is that the BV geometry used in Costa et al. [20] is tubular, which is a simplistic approach when considering that during tensile tests the veins are flattened (not inflated) when clamped by the grips. This is a common simplification found in the literature whose influence has never been evaluated [26]. There are a few studies where a more detailed vasculature model was incorporated in FEHM and compared with a reduced one, highlighting the importance of including a detailed representation of the cerebral vasculature in FE models to more accurately estimate the impact outcome such as acute SDH [32,33]. Still, the validation of the numerical models is not carried out or never considers the real geometrical shape of the veins under tensile loading, which is one of the key novelty aspects of the present work.

Therefore, the main goal of this work is to infer the influence of an accurate geometry of BV during tensile loading and create a model viable for studies where the non-linear elastic behaviour is relevant. Thus, the first goal is to evaluate the influence of the vein’s geometry in its mechanical behaviour under tensile loading, simulating the real conditions of experimental tests [24]. The second is implementing a hyperelastic model to observe its non-linear elastic behaviour. To achieve these, the BVs are precisely modelled according to the geometrical data reported by Monea et al. [24] and the simulation results compared with the experimental tensile test data. Additionally, hyperelastic constitutive models are calibrated to fit the experimental data, and the results are compared to the experiments [24] and elastoplastic approach [20].

## 2. Materials and Methods

### 2.1. FE Modelling and BV Tensile Testing in Monea et al. [24]

The experimental values used in this study were retrieved from Monea et al. [24]. These were obtained from 130 BVs, from 12 different fresh cadavers. The veins were axially stretched until failure for strain rates varying from 6.66 s^−1^ to 181.61 s^−1^.

**Figure 1:**
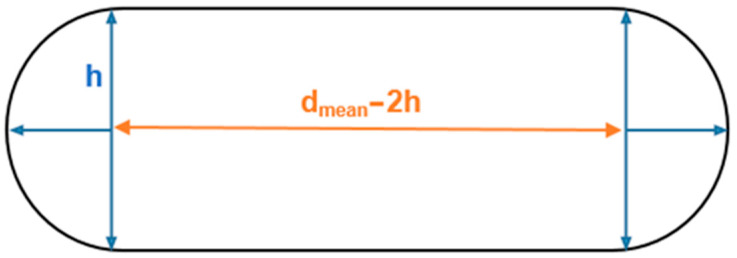
Sketch of the BVs’ cross-section based on [24].

The flattened BV shape displayed in Figure 1 is based on the data reported by Monea et al. [24]. Typically, this evidence is disregarded in the literature, and the vein structure is modelled as a circular tubular form. Even though the cross-sectional area is the same, the results may differ from those obtained in the literature due to geometrically intensified stress concentrations. This work aims to assess the influence of the fact the veins are flattened during mechanical tensile tests. Figure 1 represents the cross-sectional area of the flattened bridging vein.

Equation (1) represents the cross-sectional area of this specific geometry:

(1) $$A_{0}=[2\times h\times \left(d_{mean}-2\times h\right)+\pi \times h^{2}]$$

The values reported for the thickness (*h*) and diameter (*d_mean_*) are presented in Table 1. The diameter parameter is the average between the maximum SSS opening diameter (*Ø_max SSS opening_*) and minimum BV diameter (*Ø_min BV_*) as described by Equation (2).

(2) $$d_{mean}=\frac{{Ø}_{max\;SSS\;opening}+{Ø}_{min\;BV}}{2}$$

The structure was modelled with deformable shell elements and was sketched according to the geometry and values presented in Figure 1 and Table 1. The influence of two types of mesh regarding the type of finite element was studied: S4 and S4R shell elements. The S4 is a four-node fully integrated element, while the S4R also contains 4 nodes but is characterised by reduced integration.

In addition, a mesh convergence study was performed. A total of 6 different dimensions were used for each element type: 0.1, 0.2, 0.5, 0.7, 1, and 1.2 mm. In the case of the S4R finite element, an additional refinement was carried out due to the reduced integration, leading to an element size of 0.05 mm. These values were chosen in order to characterise a good range of obtainable data so it would be possible to select the best size for future simulations that would not need a large computational power.

Regarding the boundary conditions, on one side, all the degrees of freedom were constrained. On the opposite side, a unidirectional velocity of 1358.55 mm/s was applied. The ABAQUS 6.17 explicit solver was employed to run the calculations.

**Figure 2:**
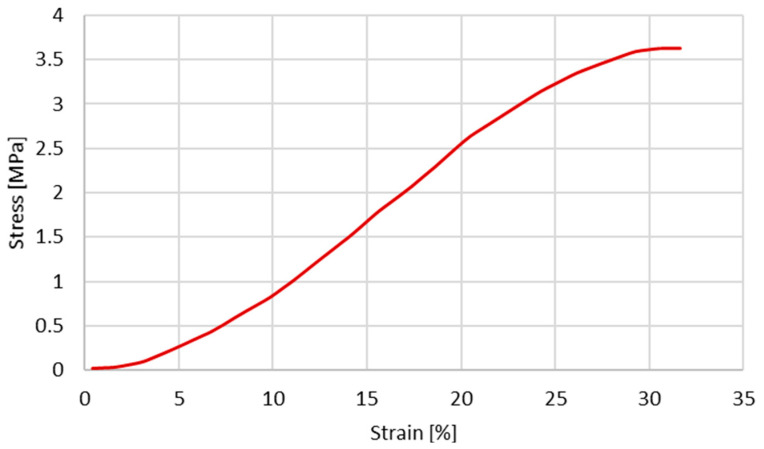
Experimental stress–strain obtained for a velocity of 1358.55 mm/s (extracted from [24]).

Since the first objective is to infer the cross-section shape influence on the stress–strain behaviour, the same constitutive model and material properties reported by Costa et al. [20] were used. The material properties used by Costa et al. [20] are presented in Table 2 and Table 3. The plastic behaviour is described based on the experimental data from Monea et al. [24], specifically in the experimental stress–strain curve (Figure 2) for an elongation rate of 136.85 s^−1^ (velocity of 1358.55 mm/s).

**Figure 3:**
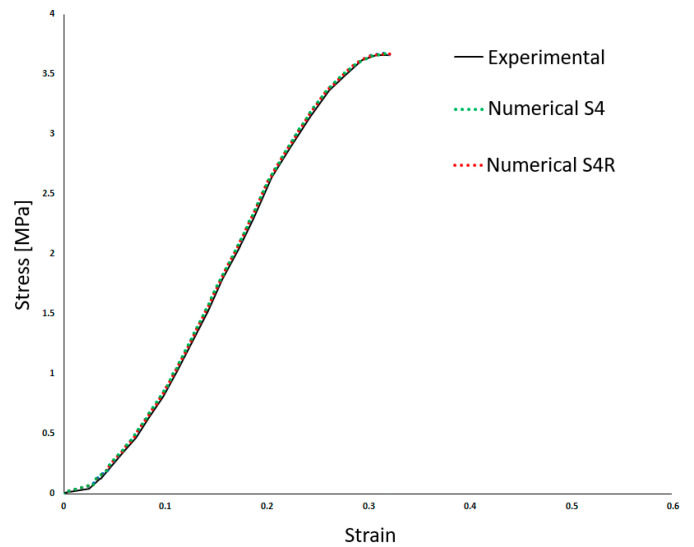
Experimental and numerical results obtained in Costa et al. [20]—stress–strain (adapted from [20]).

Table 2 shows the density (*ρ*) and the linear elastic behaviour was defined by Young’s Modulus (*E*) and the Poisson’s ratio (*ν*). Costa et al. [20] employed a high Poisson’s ratio, typical of incompressible materials such as most soft tissues. Also, a damage evolution model was implemented to simulate the BV rupture. Finally, the results from Costa et al. [20] compared to the experiments are presented in Figure 3. This makes it possible to compare both approaches in the results section.

### 2.2. Constitutive Modelling—Hyperelasticity

The final goal is to verify the applicability of the hyperelastic laws to describe the non-linear elastic behaviour of BV under dynamic tensile loading. In order to determine the potentially most accurate strain energy potential, several hyperelastic materials were considered.

For the first curve fitting approach and stability check, the following strain energy potential functions were considered: Polynomial, Reduced Polynomial, Ogden, Arruda–Boyce, Marlow, and Van der Waals [34,35,36,37]. The curve fitting was made considering only uniaxial test data. Although all revealed to be stable, there were significant deviations from the desired behaviour. Therefore, only the Marlow and reduced polynomial forms of fourth and sixth order were considered in this analysis. The form of the Marlow strain energy potential is:

(3) $$U=U_{dev}\left(\bar{I_{1}}\right)+U_{vol}(J)$$

where U is the strain energy per unit of reference volume, with $U_{dev}$ as its deviatoric part and $U_{vol}$ as its volumetric part. The $\bar{I_{1}}$ is the first deviatoric strain invariant defined as:

(4) $$\bar{I_{1}}={\bar{\lambda }}_{1}^{2}+{\bar{\lambda }}_{2}^{2}+{\bar{\lambda }}_{3}^{2}$$

where the deviatoric stretches $\bar{\lambda_{i}}=J^{-\frac{1}{3}}\lambda_{i}$; J is the total volume ratio, and $\lambda_{i}$ are the principal stretches.

The Marlow model follows an implementation based on the theory that the deviatoric strain depends only on the first invariant, $I_{1}$, which is then determined from the experimental data [38]. As a result, even though this model can, in theory, almost perfectly forecast the uniaxial tension data, the predictions of other loading modes are often far from accurate. Since, in this work, we only took uniaxial tests into account, it is still a viable model to consider.

The following equation represents the reduced polynomial strain energy function:

(5) $$U=\sum_{i=1}^{N}{C_{i0}\left(\bar{I_{1}}-3\right)}^{i}+\sum_{i=1}^{N}\frac{1}{D_{i}}{\left(J-1\right)}^{2i}$$

where U is the strain energy potential, $\bar{I_{1}}$ stands for the deviatoric strain invariant; N, $C_{i0}$, and $D_{i}$ are material parameters. The $C_{i0}$ parameter describes the shear behaviour of the material, and the $D_{i}$ parameter introduces compressibility. The N parameter is the order of the polynomial. The first term in Equation (5) is a deviatoric term that simulates the material’s shape-changing activity (angular distortion under stress). The second term is a volumetric term that models how a material’s volume changes in response to stress. The initial shear modulus, ${µ}_{0}$, and the initial bulk modulus, $K_{0}$, are given by:

(6) $${µ}_{0}=2C_{10}$$

(7) $$D_{1}=\frac{2}{K_{0}}=\frac{3(1-2\nu )}{{µ}_{0}(1+\nu )}$$

Equation (7) is valid for reduced polynomial for cases where some degree of compressibility exists (*ν* < 0.5) and *D*_1_ is not null.

In order to simulate the experimental tests from Monea et al. [24], exactly the same FE model developed in the last section was used, except the constitutive law and material properties changed. The same density of 1130 kg/m^3^ was used.

In the case of Marlow model, it is only necessary to provide the test data (Figure 2) and a form of volumetric data; in this case, the Poisson’s ratio presented in Table 2 was used. Regarding the reduced polynomial model, calibrations were performed for the 4th and 6th orders. The values of the coefficients are presented in Table 4 and Table 5, respectively.

## 3. Results

### 3.1. The Influence of the Geometrical Shape of the Cross-Section

**Figure 4:**
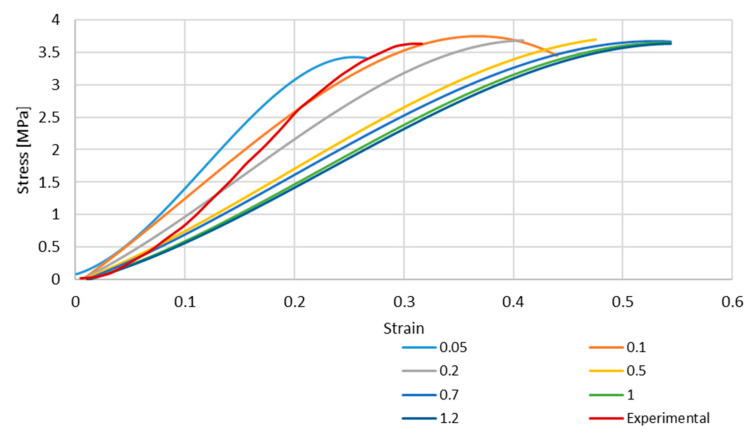
Stress–strain curve for S4R mesh for different element sizes.

**Figure 5:**
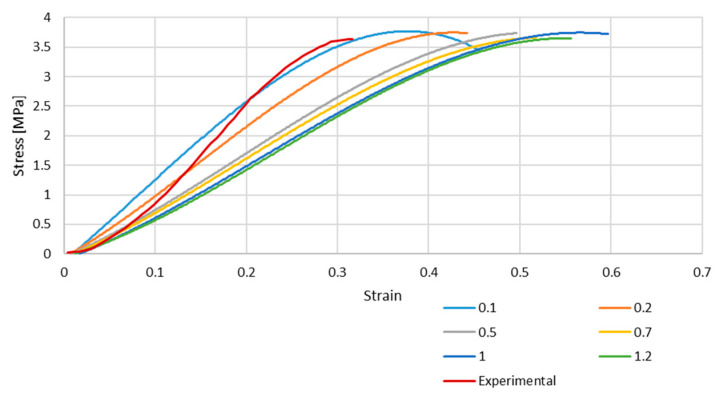
Stress–strain curve for S4 mesh for different element sizes.

Figure 4 and Figure 5 depict the stress–strain curves obtained in each simulation compared to the experimental one for S4R and S4 FE meshes, respectively. These were obtained with the model described in Section 2.1.

As it is possible to understand from the stress–strain curves presented in Figure 3, Figure 4 and Figure 5, the BV’s cross-section shape influences the mechanical behaviour compared to the results in Costa et al. [20]. It is also possible to verify that convergence was not properly achieved. However, this would need testing with smaller element sizes, and 0.05 mm is already a challenging value to employ in simulations from a computing standpoint. The element size of 0.1 mm was selected for the hyperelastic analyses due to the cost–benefit ratio considering the computational power needed and the results achieved.

### 3.2. Hyperelastic Material Model Validation

**Figure 6:**
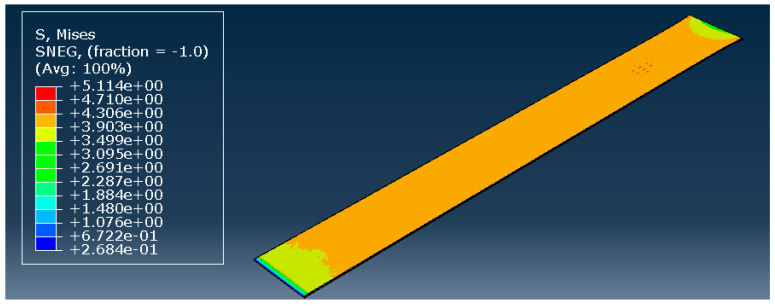
Reduced polynomial of fourth order—stress distribution [MPa].

To validate the hyperelastic model, all three most promising models during the curve fitting were used: the Marlow model, and the reduced polynomial of orders 4 and 6. The model remained precisely the same, only changing the type of material. The element size was not a variable; a constant value of 0.1 mm was used based on previous observations. The result of the simulation with the reduced polynomial of fourth order is presented in Figure 6.

**Figure 7:**
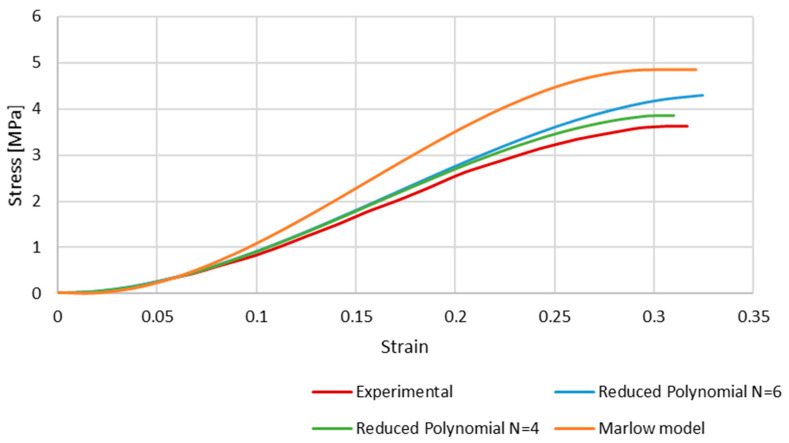
The stress–strain curves obtained with Marlow and reduced polynomial models compared to the experimental data retrieved from Monea et al. [24].

Figure 7 shows the results of the simulations, comparing the stress–strain curves obtained with each hyperelastic model and the experimental one. Although the shape of the curves is very similar, the numerical ones present a higher stress for the same strain. This is a clear phenomenon for strains approximately higher than 0.1, and is particularly significant in the case of the Marlow model. At this point, the curves stop converging but have an approximate growth rate along the strain evolution.

From Figure 7, it is possible to conclude that the Marlow hyperelastic model is the furthest from the experimental data compared to the reduced polynomial, particularly of orders 4 and 6, even though it was the closest one in the primary curve fitting described in the methodology. This phenomenon may be because curve fitting is a mathematical approach that only uses the given data to obtain the coefficients. When simulating the whole FE model, other factors, such as geometry, boundary conditions, etc., add up to the final result.

The reduced polynomial models have a smaller difference when compared to the experimental data, so we can conclude that those models would be more appropriate to use in future works.

## 4. Discussion

The study of Costa et al. [20] presents good results, which is why we based our model on it. Nevertheless, the cross-sectional shape employed was simplistic and far from the real one. In this work, we aimed to improve this aspect, maintaining, in the first part, the material model previously used, even though it is not the most viable way to study the BV’s elastic behaviour. After that step, we studied the non-linear elasticity of the veins with hyperelastic material models known for their particular mechanical behaviour and typically used to model other soft tissues.

To start, we modelled the BV with the new geometry and compared our simulation results with the experimental input. The vein was modelled using shell elements and although the next step was done with a hyperelastic approach, we started with an elastoplastic material model in order to establish a direct comparison with the previous study. It was possible to visualise that the geometry directly influences the results. Even though the cross-section area is the same in both examples, the results may vary because of the resultant forces that take action in both simulations. It is important to note that the boundary conditions were the same in this work and Costa et al. [20].

In the second half, hyperelastic material models were employed to obtain a non-linear elastic response with the new geometry. The calibration of the hyperelastic potentials used in this part was performed in ABAQUS. The three closest strain energy potentials obtained with this curve fitting were the Marlow model and the reduced polynomial of fourth and sixth orders. With these strain energy potentials, it was possible to obtain appropriate coefficients for our simulations. The results of this part of the study were satisfactory, and it is possible to conclude that the closest to the experimental data was the polynomial of the fourth order. However, one limitation must be considered since the curve plotted in Figure 2 presents a concave shape starting around 20–25%. This change in shape from convex to concave is probably related to potential micro-breaks and, therefore, loss of rigidity and the beginning of damage. This was hypothesised in our previous work [20] and in the first part of this study, where we just corrected the shape of the vein to a real one, and the cerebral BVs were modelled with elastoplastic laws (results in Figure 4 and Figure 5). However, for the second part of the study, our goal was to check the feasibility of considering hyperplastic laws to reproduce the vein behaviour. This justifies the deviations for strains from 25% to approximately 32% (failure).

## 5. Conclusions

This study was important to open this field to a new perspective on how to model these anatomic structures that, during tensile tests (or others), do not present the same structure that they present when inside the subject’s body. It was possible to conclude that the geometry of the vein structure influences the tensile stress–strain curve, which means that flattened specimens should be modelled when validating constitutive models for bridging veins.

The hyperelastic model may be relevant in the future to study specific pathologies or to the development of medical devices where the rupture of the vein is not the main property to contemplate but rather its mechanical behaviour prior to rupture. In future studies, it is compelling to model BVs as a visco-hyperelastic material model. In the literature, some studies consider viscoelastic effects more relevant, while others focus on the hyperelastic behaviour [25,26]. Therefore, it would be important to carry out a study on this topic (particularly studies where it is necessary to represent the relaxation of this type of materials, typically time-dependent). Another interesting topic for future studies would be the anisotropy of veins, as it has already been proven that the collagen fibres of the veins present different directions along the vein [10] and it would be interesting to study the influence of these different directions in FE models. Another different approach to this topic would be to model a vein divided into the three layers that compose BVs with different material properties/behaviour for each layer.

## Figures and Tables

**Table 1:** Values of the average dimensions reported by Monea et al. [24].

*Ø*_*max SSS opening*_ (mm)	*Ø*_*min BV*_ (mm)	*d*_*mean*_ (mm)	*h* (mm)
4.99 ± 1.86	1.88 ± 0.83	3.42 ± 1.18	0.044 ± 0.017

**Table 2:** Linear elastic material properties used in Costa et al. [20].

*ρ* (kg/m^3^)	*E* (MPa)	*ν*
1130	25.72	0.45

**Table 3:** Damage evolution model parameters used in Costa et al. [20].

Fracture Strain	Stress Triaxiality	Strain Rate [s^−1^]	Displacement at Fracture
0.31875	0.33	135.86	0.05

**Table 4:** Coefficients used for the reduced polynomial of 4th order. *D*_2_ to *D*_4_ are negligible.

*C*_10_ (MPa)	*C*_20_ (MPa)	*C*_30_ (MPa)	*C*_40_ (MPa)	*D*_1_ [MPa^−1^]
0.507231065	21.4170360	−92.7419760	142.420876	0.203947043

**Table 5:** Coefficients used for the reduced polynomial of 6th order. *D*_2_ to *D*_6_ are negligible.

*C*_10_ (MPa)	*C*_20_ (MPa)	*C*_30_ (MPa)	*C*_40_ (MPa)	*C*_50_ (MPa)	*C*_60_ (MPa)	*D*_1_ [MPa^−1^]
0.464417032	29.4654136	−278.559124	1660.99772	−5202.69082	6384.20622	0.222748669

## Data Availability

Data are contained within the article.
